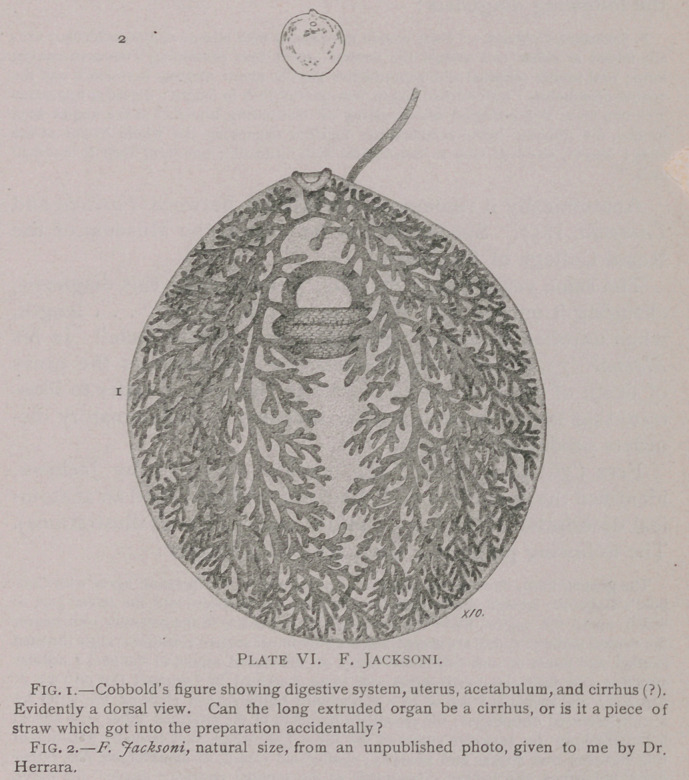# The Anatomy of the Large American Fluke (Fasciola Magna) and a Comparison with Other Species of the Genus Fasciola, S.ST.

**Published:** 1895-03

**Authors:** Chas. Wardell Stiles

**Affiliations:** Zoölogist, Bureau of Animal Industry


### THE ANATOMY OF THE LARGE AMERICAN FLUKE (*FASCIOLA MAGNA*) AND A COMPARISON WITH OTHER SPECIES OF THE GENUS FASCIOLA, S.ST.

BY CHAS. WARDELL STILES, PH.D.,
ZOÖLOGIST, BUREAU OF ANIMAL INDUSTRY.

CONTAINING ALSO A LIST OF THE CHIEF EPIZOÖTICS OF FASCIOLIASIS (DISTOMATOSIS) AND A BIBLIOGRAPHY OF FASCIOLA HEPATICA.

BY ALBERT HASSALL, M.R.C.V.S.

(Continued from vol. xv., 1894, page 462.)

### III. Fasciola gigantea Cobbold, 1856.

PLATE V.

SYNONYMY:

1856, *F. gigantica* Cobbold.
1858, *Distomum giganteum* Diesing.
1858, *Distoma hepaticum* ex p. Gerv. et van Ben.
1859, *Fasciola gigantea* Cobbold.
1892, *Cladocœlium giganteum* ex p. Stossich.

HOST:

Giraffe (*Giraffa camelopardalis*), Liver.

GEOGRAPHICAL DISTRIBUTION: Found but once. England, in a giraffe belonging to a travelling menagerie.

### LITERATURE.

BRAUN, Max—

'93.—Vermes; Bronn's Klassen und Ordnungen des Thier-Reichs, IV, 1.

COBBOLD, T. Spencer—

'54.—On the Anatomy of the Giraffe (*Camelopardalis giraffa*, Linn.); The Annals and Mag. of Nat. Hist., 2d ser., Vol. XIII, pp. 484–488.

'55a.—Description of a New Species of Trematode Worm, with Observations on the Structure of Cercariæ; Proc. Royal Physical Soc. of Edinburgh. Vol. I (1854–1858), 1858, p. 9 (presented Jan. 24, 1855).

'55b.—Description of a New Species of Trematode Worm (*Fasciola gigantica*); The Edinburgh New Phil. Journn. New Ser. Vol. II, pp. 262–267.

'56.—Description of a New Species of Trematode Worm (*Fasciola gigantica*); Report of the 25th Meeting of the British Association for the Advancement of Science (at Glasgow), 1855, Notices and Abstracts of Miscellaneous Communications to the Sections, pp. 108–109. London, 1856.

'60.—Synopsis of the *Distomidæ;* Journ. of the Proc. Linnean Soc. of London (for 1859) Zool. Vol. V, pp. 1–56, 1860.

'64.—Entozoa; An Introduction to the Study of Helminthology. London.

DIESING—

('58.)—Revision der Myzhelminthen; Sitzungsber. der K. Acad. XXXI.

GERVAIS, Paul, et van BENEDEN—

('58.)—Zoologie Médicale, Paris.

LEUCKART, Rudolf—

('63.)—Die Parasiten des Menschen. 1 Aufl.

'79–'93.—Die Parasiten des Menschen. 2te Aufl.

SONSINO, Prospero—

'90.—Studie notizie elmintologiche; Proc. verb. d. società Toscana di scienze natur. 4 maggio, 16 pgs. Ref. (Braun) C. f. B. u. P. VIII, p. 309.

STILES—

'94–'95.—The present paper.

### HISTORICAL REVIEW.

COBBOLD ('54, 485) discovered several specimens of flukes of the genus *Fasciola* in the liver of a giraffe during a post-mortem examination nine days after the death of the animal. He communicated this find, according to a footnote, to the Royal Physical Society of Edinburgh on April 5, 1854. In his printed account he also mentions finding "cysticerci" in the same liver. He ('55a) presented an account of the anatomy of the worms before the same society on January 24, 1855. This time, as the title of his paper shows, he speaks of finding "cercariæ" as well. In the proceedings of the Society only a short abstract appears in type. He afterward (September, 1855) presented a paper before the British Association, and evidently published the same, with very slight changes, in two places. He states ('55b) that no less than forty specimens were washed out by means of a syringe. The diagnosis reads:

*Fasciola gigantica* Cobbold. Corpore compresso, elliptico-lanceolato, tres uncias longo, antrorsum attenuato; ore hausterioque antice; collo elongato, cylindrico; cauda rotundata; ventriculo dendritico, ramis clausis.

Habitat in hepate Camelopardalis giraffæ.

The parasite varies in length from 1½ to 3 inches, averaging about 2 inches; the breadth averages 3 lines, but may be one-third of an inch; substance of the body is thinner than that of *F. hepatica;* anterior extremity prolonged forward, oral sucker half a line in diameter. Œsophagus short; 8 to 10 lateral branches to each longitudinal intestinal sac. Excretory system consists of a single median trunk which sends branches upward and toward the sides; this system Cobbold looks upon as a circulatory system, and quotes M. Blanchard's opinion that the caudal opening results from "over-distention of the canal, which readily gives way at this its weakest point; our own (*i. e.*, Cobbold's) attempts to inject have confirmed this observation." Other organs "resemble in all respects those seen in" *F. hepatica*.

PLATE V. F. GIGANTEA.

FIG. 1.—Ventral view, showing digestive system and acetabulum.

FIG. 2.—Dorsal view, showing excretory system. Cobbold's figures, '64.

He then speaks in an appendix of "two kinds of cericaria found associated with the above-described trematode. One group of these cysts infested the liver, where they appeared either at the surface in the form of small, hard, projecting points, or were thinly scattered throughout the substance of the gland. They were very numerous, and some had undergone calcareous degeneration."

The others consisted of "semi-transparent cysts in the cellular aponeurosis surrounding the stylo-glossi and lingualis muscles."

It is impossible to decide definitely, either from his description or figures, just what these "cercariæ" or "cysticerci" are, although one would naturally think of *Cysticercus bovis* in connection with the form found in the tongue-muscles.

In his author's abstract ('56) the account is almost exactly the same as the one just reviewed, except that "*teruncìatum longo*" appears in place of "*tres uncias longo*" in the diagnosis, and the appendix and figures are not given.

GERVAIS and van BENEDEN ('58, II, p. 201, not at my disposal), according to Cobbold, consider *F. gigantica* identical with *F. hepatica*.

DIESING ('58, p. 28, pp. 131–132, not consulted) changes the name to *Distomum giganteum*.

COBBOLD ('60, p. 4) then accepts the name *Fasciola gigantea* with the diagnosis:

"Corpus planum oblongum, lanceolatum, antrorsum attenuatum, retrorsum obtusum. *Collum* elongatum cylindricum. *Os* terminale, anticum. *Acetabulum* ore majus, superum ad colli basin. Longit. 1–3 unc.; latit. ¼–½ unc. . . . There existed in the liver (*i. e.*, of the giraffe) a number of Cysticerci; as well as three larval Distomes in cysts connected with the sublingual cellular aponeurosis."

LEUCKART ('63, I, p. 30, not consulted), according to Cobbold ('64), examined two specimens of the worm, and claims that the species "*Distomum giganteum*" is perfectly distinct from "D. hepaticum."

COBBOLD ('64, pp. 161–162, frontispiece, two figs.) reproduces his original figures, greatly enlarged. He makes a comparison between *F. gigantea* and *F. hepatica*, and concludes that the number of secondary branches in the intestines and excretory system of the former is greater than in the latter. The former is also larger than the latter; in form there is also a difference, in that the lower half of the body of *F. hepatica* is gradually narrowed toward the caudal point, presenting a more or less V-shaped outline; in *F. gigantea*, on the other hand, the narrowing commences very near the caudal end, the latter being bluntly curved or even truncated.

LEUCKART ('79–'94, I, 2. Abth., p. 179) accepts "*Dist. giganteum*" as a good species, and places this form with *D. Jacksoni* and *D. hepaticum* in the first subdivision (*Fasciola* Linn.) of the genus *Distomum*.

SONSINO ('90, original not obtainable here, I quote from Braun's review) seems inclined to believe that *D. magnum* and *D. giganteum* are perhaps identical.

BRAUN ('93, p. 910, Pl. XXI, 2) places *D. giganteum* in the sub-genus *Cladocœilum*, and copies one of Cobbold's figures.

Desiring to compare *F. gigantea* with *F. magna*, I have addressed letters at several different times to the Director of the Hunterian Museum, of London, asking for the loan or exchange of one or more specimens. As no response has ever reached me, I communicated with my former teacher, Geheimrath Leuckart, and asked his opinion as to the relation of the two species to each other. He replied to the effect that Cobbold's species, *F. gigantea*, is unquestionably distinct from Bassi's *D. magnum*—in fact, it resembles *D. hepaticum* more closely than it does *D. magnum;* nevertheless, that *F. gigantea* represents a true species. (See postscript at end of article.)

The species is evidently insufficiently described, and can be accepted only on Leuckart's authority.

I regret that on account of my inability to obtain specimens of *F. gigantea* I am not in a position to make any original statements in regard to its anatomy. From the above historical review, however, we may accept the following as a provisional specific diagnosis:

### SPECIFIC DIAGNOSIS.

*F. gigantea* Cobbold, 1856. Body flat, oblong, lanceolate, 75 mm. long, 3–12 mm. broad; anterior extremity attenuate, posterior extremity obtuse; neck cylindrical; oral sucker terminal, 1.12 mm. in diameter; œsophagus extends nearly to the acetabulum; 8 to 10 lateral branches to each longitudinal intestinal sac; acetabulum larger than mouth; other organs agree with those of *F. hepatica*.

Diagnosis based upon Cobbold's description of specimens, which were taken nine days after the death of the host, and which, therefore, must have been more or less macerated.

### IV. Fasciola Jacksoni Cobbold, 1869.

PLATE VI.

SYNONYMY:

1847, *Distoma hepaticum* Jackson.
1858, *D. elephantis* Dies. sp. inq.
1860, *D. elephantis* Cobbold.
1869, *Fasciola Jacksoni* Cobbold.
1892, *Cladocœlium elephantis* Stossich.
1893, *Distomum Jacksonii* Braun.

HOST:

*Elephas indicus*, gall-ducts.

GEOGRAPHICAL DISTRIBUTION: *North America:* Boston, Mass. (Jackson), elephant imported from East India. *South America:* Buenos Ayres, Argentine Republic (Wernicke and Herrera), in imported elephant. *Asia:* British India, Burmah (Rangoon by Thacker, Hawkes); Dominion of Hyderabad (Secunderabad by Adams).

### BIBLIOGRAPHY.

BRAUN, Max—
'93.—Vermes, pp. 376, 910; Bronn's Klassen und Ordnungen des Thier-Reichs.

COBBOLD, T. Spencer—
'60.—Syn. *Distomidæ:* Journ. of the Linnean Soc. of London for 1859. Zool. Vol. V. (1860).
'69a.—Description of a species of Trematode from the Indian Elephant, with Remarks on its Affinities; Quart. Journ. of Micr. Sc., Jan., pp. 48–49.—Descrizione di una specie die Trematode dell' elefante Indiano, con avvertenze intorno alle sue affinita. Tradotto per L. L.; Giorn. di Anat., Fisiol. e Patolog. degli animali. Anno 1, 1870, pp. 47–49.
'69b.—Supplement to Entozoa, p. 80.
'73.—Manual of the Parasites of our Domesticated Animals, p. 13.
'79.—Parasites: A Treatise on the Entozoa of Man and Animals. London.
'82.—The Parasites of Elephants; Trans. of the Linnean Soc. of London, 2d ser., Zool., Vol. II, Part 4, pp. 223–258, Plates XXIII–XXIV.

DIESING—
'58.—Revision der Myzelminthen; Sitzungsber. d. Math. Nat. Kl. d. K. Akad. d. Wissensch., Bd. XXXII.

FITZ, R. H.—
'76.—Anatomy of the *Fasciola Jacksoni* Cobbold; N. Y. Med. Journ., Nov., 1876, XXIV, 5, pp. 513–518.

JACKSON—
'47.—A Descriptive Catalogue of the Anatomical Museum of the Boston Society for Medical Improvement. Boston.

V. LINSTOW—
'78.—Compendium der Helminthologie. Hanover.

STILES—
'94–'95.—The present paper.

### Historical Review.

Jackson ('47, p. 322) determined as *Distoma hepaticum* some flukes found in 1835 in the gall-ducts of a young East Indian elephant which died in Boston. Later authors state that Jackson records these worms from the duodenum, as well as from the gall-ducts; but this is erroneous, as Jackson writes:

". . . immense numbers were found in the ducts, and of the ascarides a few were found in the duodenum. In the same jar are several larvæ from the stomach and duodenum. There was ascites, with disease of the liver, and in the stomach near the pylorus a large, deep, chronic ulcer, of a circular form and perfectly defined. (No. 908.)"

No anatomical observations were made.

2

I

Plate VI. F. Jacksoni.

Fig. 1.—Cobbold's figure showing digestive system, uterus, acetabulum, and cirrhus (?). Evidently a dorsal view. Can the long extruded organ be a cirrhus, or is it a piece of straw which got into the preparation accidentally?

Fig. 2.—*F. Jacksoni*, natural size, from an unpublished photo, given to me by Dr. Herrara.

Diesing ('58, p. 50) mentions the parasite found by Jackson as *D. elephantis*. No description is given, so that the specific term *elephantis* cannot be accepted.

COBBOLD ('60, p. 9) also mentions the worm under the same name. Later ('69a, pp. 48–49) he received two specimens of flukes taken by Vet. Surg. J. Thacker, Madras Army, from the liver of an elephant in Rangoon. The English helminthologist recognized the form as identical with fifteen specimens which Huxley had a short time previously exhibited in his lectures before the Royal College of Surgeons. Cobbold surmised that thep arasites are identical with the flukes recorded by Jackson ('47), and describes them under the name *Fasciola Jacksoni* with the following diagnosis:

F. Jacksonii, Cobbold.—" Body armed throughout with minute spines, orbicular, usually folded at either end toward the ventral aspect, thus presenting a concavo-convex form; oral sucker terminal with reproductive papilla about midway between it and the ventral acetabulum; intromittent organ one-quarter inch in length; digestive apparatus with two main zigzag-shaped canals, giving off alternating branches at the angles thus formed, the ultimate cæcal ramifications together occupying the whole extent of the body; length, one-half inch to five-eighths inch; breadth, one-third inch to one-half inch."

Anatomically it represents a transition between *Fasciola* and *Campula*, 1857. Specimens can be seen in the Museum of the Royal College of Surgeons.

The same year ('69b, p. 80, Fig. 3) he reprints this diagnosis, changing it only in one place, so that it reads: ". . . length, when unrolled, from one-half inch to five-eighths inch." In his *Manual* ('73, p. 13) Cobbold refers to *F. Jacksonii* as the cause of death of some of the elephants in Burmah, in order to illustrate that larger animals readily succumb to inflammatory disorders produced by flukes.

FITZ ('76) then studied the material recorded by Jackson, identified the specimens as *F. Jacksoni*, and published an anatomical description of the same (unfortunately without illustrations). The following presents a summary of his results:

The general form, dimensions, and the arrangement of the intestine agree with Cobbold's diagnosis; genital papilia absent, in its place a depression, into the lower part of which opens the vagina, while the opening for the penis is in the posterior wall nearer the ventral surface; spines are absent, but the abdominal surface possesses ridges directed caudad, and traces of such ridges are found on the dorsal aspect of the neck; cellular structure and muscular bands agree with those described by Leuckart (1st ed.) for *F. hepatica;* oral sucker is followed by a voluminous bottle-shaped pharynx, the latter by a short crenated œsophagus; the secondary branches of the intestinal tubes arise almost invariably from he *posterior* aspect of the tubes from which they branch; post-buccal ring between oral sucker and pharynx; shell-gland round, of considerable size, rather dorsal in position and behind (=caudad) of the seminal vesicle; "above" the ootyp "communicates by a short, narrow tube with the conjoined yolk and ovarian ducts;"

ovaries ventral, a short distance behind the ventral sucker, and are two "large convoluted tubes with blind projections. These tubes unite near the median line into a single tube or duct, which passes upward toward the front of the shell-gland, becomes very narrow, and at one point sharply constricted. Into this narrow duct beyond the constriction enters the vagina" (Laurer's canal) "and the tube then unites with the yolk-duct, forming a T-shaped figure, the lower arm of which enters the gland." The yolk-glands agree with those of *F. hepatica*, the yolk-duct "uniting with the ovarian duct." The convolutions of the uterus lie rather behind (—caudad) and around the ventral sucker testicles agree in general with those of *F. hepatica*. Author believes copulation takes place through Laurer's canal.

(*To be Continued.*)

## Figures and Tables

**Plate V. f1:**
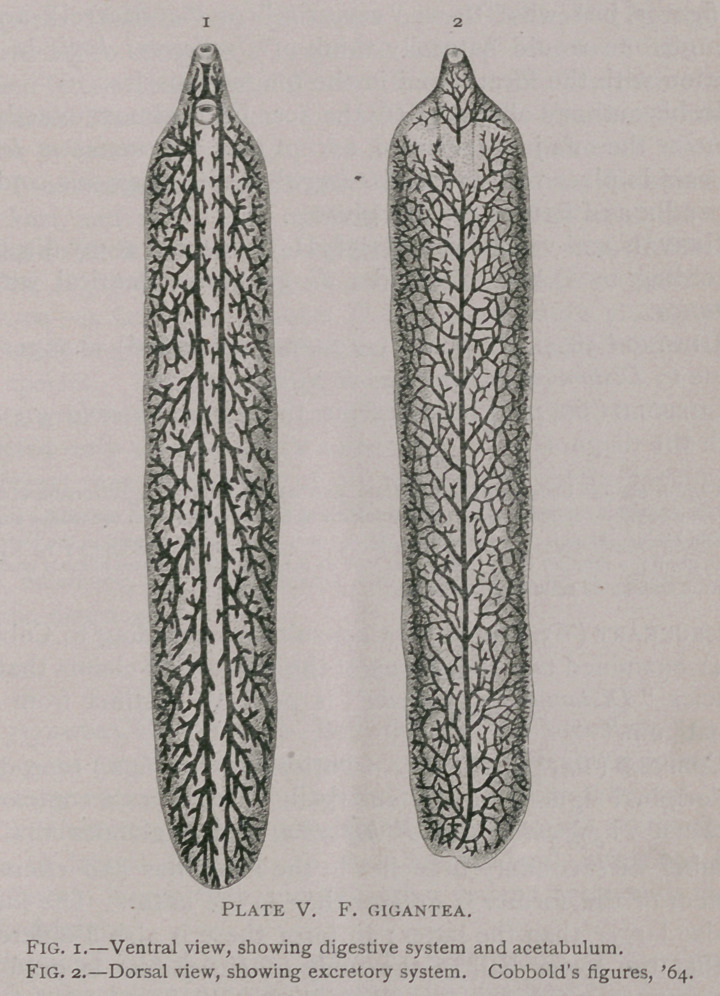


**Plate VI. f2:**